# B cells intervention in inflammatory mechanism of ankylosing spondylitis: A visualization analysis for the past 20 years

**DOI:** 10.1097/MD.0000000000035904

**Published:** 2023-11-17

**Authors:** Qing Yu, Zhaoyi Liu, Xiaohan Xu, Hongxiao Liu

**Affiliations:** a Guang’anmen Hospital, China Academy of Chinese Medical Sciences, Beijing, China; b Beijing University of Chinese Medicine, Beijing, China.

**Keywords:** ankylosing spondylitis, B cells, CiteSpace, inflammatory mechanism, visualization analysis

## Abstract

Ankylosing spondylitis (AS) is an autoimmune disease with complex inflammatory mechanism. The aim of this study is to apply the methods of bibliometrics and knowledge mapping to analyze the research trends and hot spots of B cells intervention in inflammatory mechanism of AS. Global published articles on B-cells intervention in inflammatory mechanism of AS were retrieved from the Web of Science (WOS) database from 2004 to 2023. CiteSpace 6.1.R6 software was used to conduct the visualization analysis of countries, authors, institutions, references and keywords in this field. A total of 359 related articles were collected. Since 2004, the number of articles published in the field of B cells intervention in inflammatory mechanism of AS has shown a fluctuating upward trend. The 29 core authors are part of a research group centered on Bowness, Paul and Breban, Maxime. The main research institutions are Anhui Med Univ and Charite. Co-citation analysis reveals that research in this field is currently focused on “intergenic region” and “bone mineral density.” Keyword analysis shows that the current research hotspots and trends in this field mainly focus on the cellular immune mechanism, humoral immune mechanism and clinical application value of B cells intervention in inflammatory mechanism of AS. In the past 20 years, the research on the mechanism of B cells intervention in AS inflammation has focused on B cells intervention in AS inflammation through humoral and cellular immune mechanisms. The future research focus may tend to use B cells as a new therapeutic target for AS.

## 1. Introduction

Ankylosing spondylitis (AS) is an autoimmune systemic disease characterized by inflammatory disorders of the spine and sacroiliac joints. Low back pain is the most common and typical symptom of AS. At the end of the disease course, hip and spine ankylosis may be caused, resulting in limitation of motor function and serious impact on normal life of patients. The main pathological features of AS are inflammation and bone destruction of lumbar, cervical, and thoracic spinal joints and ligaments and sacroiliac joints, leading to heterotopic ossification.^[1]^ Autoimmune-mediated inflammation is the initial step in the development of AS, so early anti-inflammatory therapy is important to delay the progression of bone destruction and reduce the rate of disability.

Inflammatory response of AS is often associated with B lymphocyte cellular and humoral immune dysfunction. B1 cells, as innate immune cells, can produce polyreactive autoantibodies, which can be deposited in tissues, induce complement dependent cytotoxicity by activating complement, or cause tissue damage by antibody-dependent cell-mediated cytotoxicity. It can also activate immune cells through the dual action of B cell receptors and toll-like receptors located on B cells to induce inflammatory response. T follicular helper cells can contribute to the humoral immune response by interacting with homologous B cells, promoting the differentiation of B2 cells into plasma cells and producing high-affinity antibodies. The interaction between pathological T cells and B cells, as evidence of affinity maturation of autoantibodies, is closely related to the pathogenesis of autoimmunity. In addition, B cells can produce proinflammatory cytokine such as interleukin-6(IL-6) and interleukin-2(IL-2). IL-6 can promote the proliferation of B cells and exert a pluripotent effect on T cells and other types of cells, an inflammatory response that promotes autoimmunity.^[1,2]^ Studies have shown that high expressions of B cell activating factors in autoimmune diseases such as AS may lead to loss of self-tolerance by autoreactive B cells and the development of immune pathological lesions.^[2]^

Bibliometrics can quantitatively describe and visualize the key features of published literature in a certain field and has been widely used in various fields.^[3]^ However, in the case of AS, more bibliometric studies focus on the clinical diagnosis, treatment and pathological changes of AS, while few studies on the inflammatory mechanism in its pathogenesis. As an indispensable part of AS autoimmunity, B cells play an important role in mediating the inflammatory response of AS. However, the literature in this field is not comprehensive and the literature content is complicated, which makes it difficult for researchers to quickly obtain, screen and summarize the research results on the mechanism of B cells intervention in inflammatory mechanism of AS. Therefore, based on bibliometrics, this study conducted a visual analysis of the countries, authors, institutions, references and keywords included in the literature on the mechanism of B cells intervention in AS inflammation, aiming to show the research trends and hot spots in this field and provide references for subsequent scientific research and clinical development.

## 2. Materials and Methods

### 2.1. Data retrieval and collection

The data were from the Web of Science (WOS) database. The retrieval strategy was: TS = (Ankylosing spondylitis OR axial spondyloarthritis OR non-radiographic axial SpA OR nr-axSpA) AND TS = (B cells OR Lymphocyte “B.” OR Bone marrow Lymphocyte OR Plasma cells). The retrieval period was from January 2004 to January 2023.The types of articles were selected as journals articles and dissertations, and the language was English, and 697 articles were obtained. A total of 359 articles were obtained after manual screening to exclude those whose keywords or abstract contents did not meet the requirements.

### 2.2. Data analysis

The included articles were exported in TXT format, which contained information such as journal sources, titles, authors, research institutions, abstracts, keywords and so on. CiteSpace is widely used bibliometric analysis software that captures timely and accurate developments in the discipline and helps researchers to quickly access useful scientific information.^[1]^ CiteSpace 6.1.R6 software selected the time slice as 1 year, and visualized the data with countries, authors, institutions, co-cited articles and keywords as nodes.

## 3. Results

### 3.1. Trends in publications

**Figure 1.**
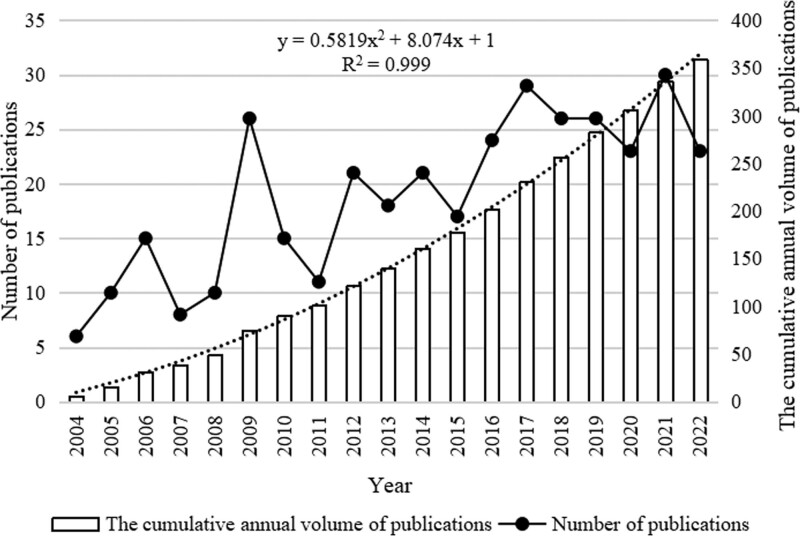
Trends in annual publications about B cells intervention in inflammatory mechanism of AS. AS = ankylosing spondylitis.

Based on the data collected, the annual output of the 359 articles included in the study was calculated (Fig. 1). From 2004 to 2023, the total volume of publications showed a fluctuating upward trend, with an annual average of 18.89 articles, indicating the development of research in this field. From 2007 to 2009, it was a stage of rapid development, with an annual average of 14.67 articles. From 2017 to 2020, the number of publications was still at a high level, with an average of 26 articles per year, but showed a decreasing trend, and it peaked in 2021, with a total of 30 articles. The content of articles mainly included clinical research, animal experiment and literature research.

### 3.2. Analysis of countries

**Figure 2.**
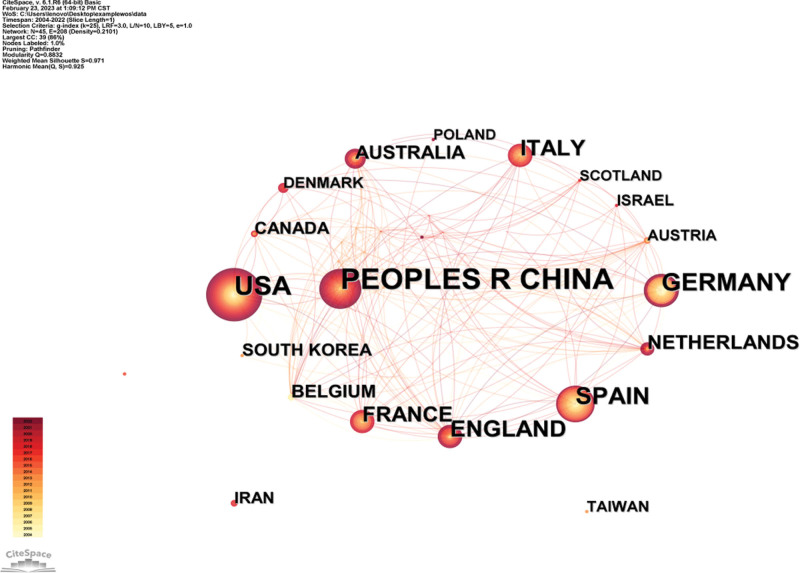
Cooperation network among countries.

Taking the country as the node for the visualization analysis (Fig. 2), there are 45 nodes and 208 lines in the atlas. The density of the atlas is 0.2101. There are 13 countries with the volume of 10 publications or more. China, the United States and Germany rank the top 3, accounting for 23.68%, 15.32% and 11.14% of the total number of publications respectively. These results suggest that these 3 countries play an important role in the study of B cells intervention in inflammatory mechanism of AS. The country visualization shows close cooperation between countries,^[4]^ and as shown in Figure 2, the level of international cooperation among China, the United States, Germany, and Spain is higher.

### 3.3. Analysis of authors

**Figure 3.**
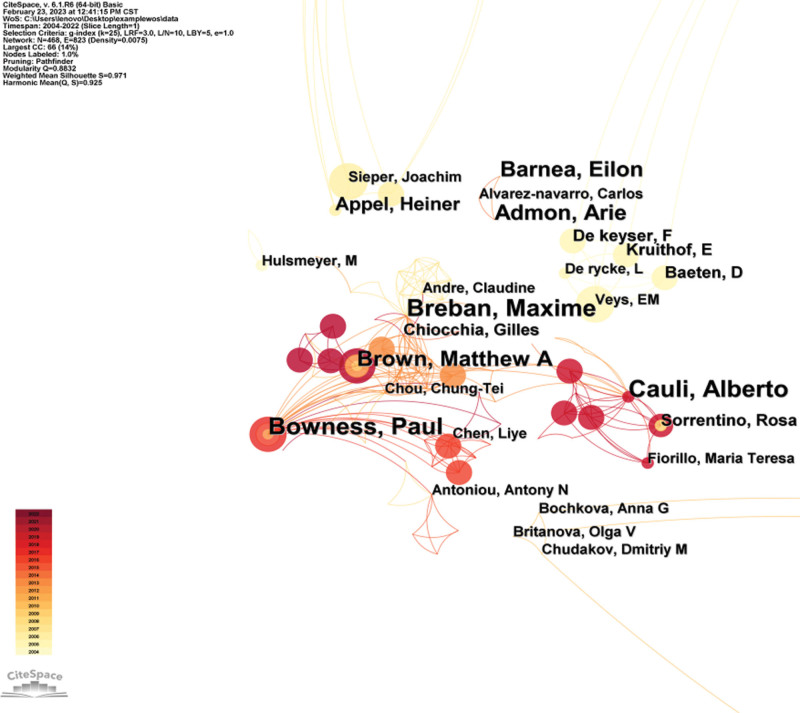
Cooperation network among authors.

A visualization analysis is performed with the author as the node (Fig. 3), and the atlas is composed of nodes and links. The size of the nodes is positively correlated with the volume of their posts, and the lines represent the cooperative relationship between the authors. The thicker the lines, the closer the cooperation between the 2, and the more lines, the stronger the centrality.^[5]^ There are 468 nodes and 823 links in the atlas. The density of the Atlas is 0.0075. There are 12 authors with more than 4 publications. The top 3 are Bowness, Paul, Breban, Maxime, Cauli, Alberto, and with these 3 authors as the center form a close-knit group. Among them, the largest collaboration scale and the highest network structure is Bowness, Paul team, which published a great number of articles about B cells intervention in inflammatory mechanism of AS and has great impact in this field. According to the Price Law,^[6]^ we can calculate the minimum number of publications of core authors, and those whose number of publications is greater than or equal to 3 belong to core authors, totaling 29, accounting for 6.20% of the total number of authors. The total number of publications of core authors is 124, accounting for 34.54% of the total.

### 3.4. Analysis of institutions

**Figure 4.**
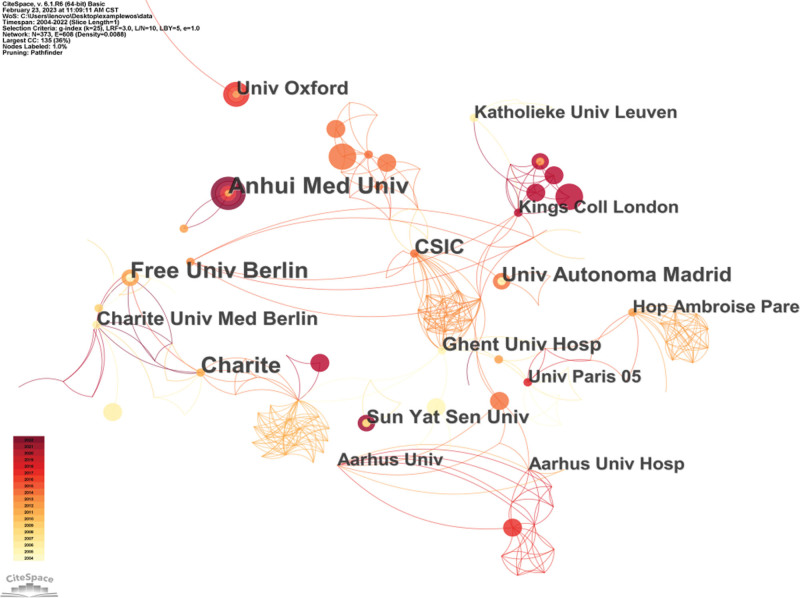
Cooperation network among institutions.

A visualization analysis is performed with the institution as the node (Fig. 4). The graph has 373 nodes and 608 lines. The density of the graph is 0.0088. A total of 373 institutions contributed to the number of AS publications and there are 16 institutions with more than 5 publications. The top 5 institutions are Anhui Med Univ (10 articles), Charite (9 articles), Free Univ Berlin (9 articles), Univ Autonoma Madrid (7 articles), and CSIC (7 articles), formed by Anhui,China, Berlin,Germany, Madrid,Spain as a representative of the 3 more influential regional cooperation center. Among these, Ghent Univ Hosp has the highest intermediary centrality (0.16), highlighting its central role in the exchange of information. There is close cooperation among the various institutions and the network structure of research institutions is formed preliminarily.^[5]^

### 3.5. Analysis of highly co-cited references

**Figure 5.**
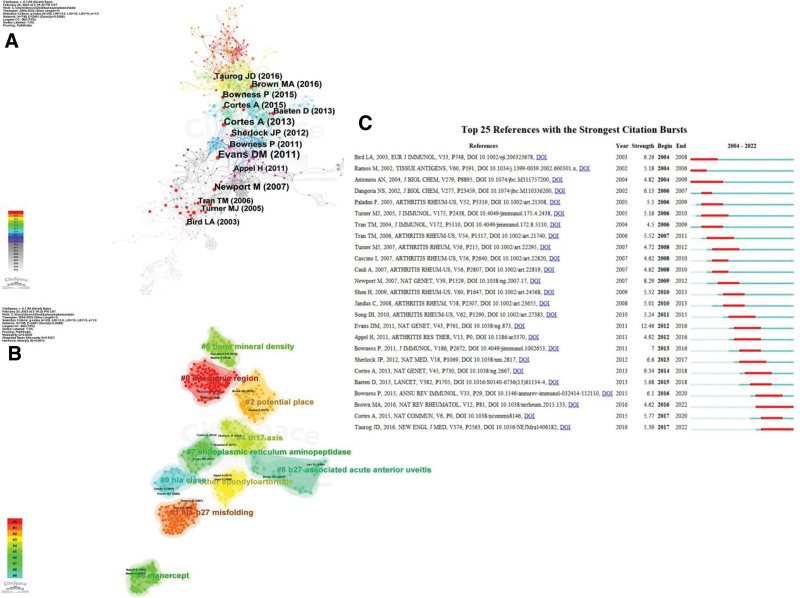
Analysis of co-cited references. (A) Co-occurrence diagram of co-cited references. (B) Cluster diagram of co-cited references. (C) Burstiness analysis of co-cited references.

Co-cited references refer to the references cited by 2 (or more) articles at the same time, indicating the close relationship between the articles. The more total citations of the reference, the higher the influence of the reference.^[5]^ CiteSpace 6.1.R6 software was used to analyze co-cited references for co-occurrence (Fig. 5A), clustering (Fig. 5B) and salience (Fig. 5C). A total of 8 articles are cited more than 15 times, of which the highest cited frequency is 30 times. The top 10 clusters with the largest range are #0 intergenic region, #1 hla-b27 misfolding, #2 potential place, #3 other spondyloarthritide, #4 th17 axis, #5 bone mineral density, #6 etanercept, #7 endoplasmic reticulum aminopeptidase, #8 b27-associated acute anterior uveitis and #9 hla clase. In addition, Figure 5C shows the top 25 references with the strongest citation bursts, showing the evolution of citations in this field over time. We find earlier studies in B cells intervention in inflammatory mechanism of AS including “hla-b27 misfolding”，“etanercept” and “b27-associated acute anterior uveitis.” Researches in this field are currently focused on “intergenic regions,” “bone mineral density” and “potential places.”

### 3.6. Analysis of keywords

**Figure 6.**
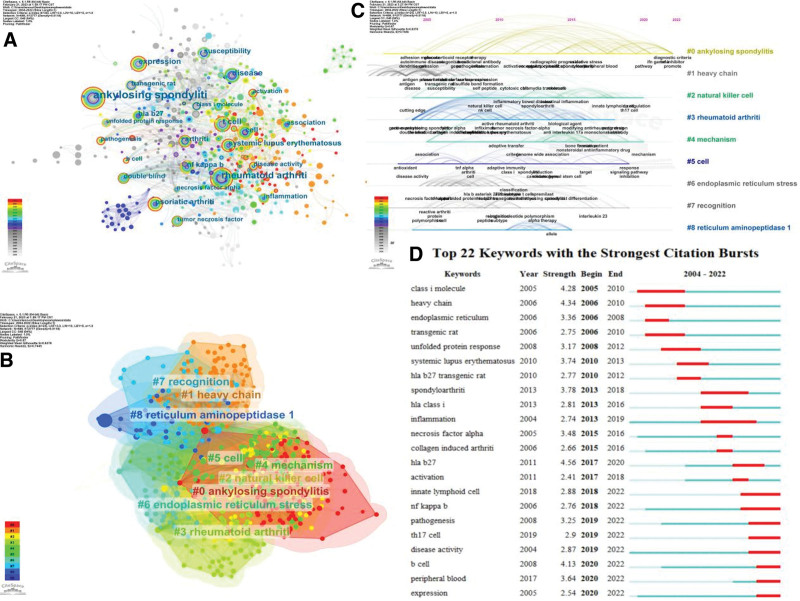
Analysis of keywords. (A) Co-occurrence diagram of keywords. (B) Cluster diagram of keywords.(C) Timeline diagram of keywords.(D) Top 22 keywords with the strongest citation bursts.

The co-occurrence analysis of keywords is helpful to understand the current situation and research hotspots in this field. Through the co-occurrence map (Fig. 6A), we can see that there are 688 nodes, 2777 lines, and the density is 0.0118. There are 12 keywords with more than 30 frequencies, and the top 10 are ankylosing spondyliti (240 frequencies), rheumatoid arthriti (95 frequencies), disease (49 frequencies), T cell (47 frequencies), expression (40 frequencies), cell (39 frequencies), psoriatic arthriti (38 frequencies), systemic lupus erythematosus (35 frequencies), arthriti (32 frequencies), nuclear factor-κB (32 frequencies). Centrality can reflect the degree of closeness between keywords. The higher the centrality, the wider the scope of articles involved in this keyword.^[6]^ The top 3 are T cell, double blind and rheumatoid arthriti, followed by genome wide association, autoimmune disease, arthriti, expression, unfolded protein response, susceptibility, psoriatic arthriti. It can be concluded that most articles in this field focus on T cell, double blind and rheumatoid arthriti.

CiteSpac 6.1.R6 software carries on k-means clustering analysis to keywords (Fig. 6B), which can reflect the relationship between important nodes and the research status of the field. 9 clusters with the largest range are selected for analysis. Among them, the largest cluster is #0 ankylosing spondylitis, followed by #1 heavy chain, #2 natural killer cell, #3 rheumatoid arthritis, #4 mechanism, #5 cell, #6 endoplasmic reticulum stress, #7 recognition, #8 reticulum aminopeptidase 1. In the cluster atlas, the overlap degree of each cluster module is high, which shows that the relationship among clusters is relatively close. To sum up, it can be divided into 4 groups: #3, #6, #8 are endoplasmic reticulum secreted proteins involved in the construction of AS inflammatory signaling pathway; #2 and #5 represent that other cells cooperate with B cells to participate in the inflammatory mechanism of AS; #0 and #3 represent that B cells participate in the inflammatory mechanism of other diseases; #4 and #7 are mainly the recognition mechanism of the immune response of AS, etc.

CiteSpace 6.1.R6 software carries on a time-line analysis of keywords (Fig. 6C). By studying the evolution of keywords over time, we can see the gradual transformation of keywords from the early“class i molecule,” “heavy chain”and“endoplasmic reticulum”to“spondyloarthritide,” “hla class i”and“inflammation.” In the past 2 years, there have been some new hot keywords, such as“B cell,” “peripheral blood,” “expression.”

Keyword salience is to detect a large change in the number of citations in a certain period of time, which can predict the development trend of the research field. Keywords are analyzed according to the year of emergence, and the period from 2004 to 2023 is chosen as the minimum period of 2 years to detect the emergent words (Fig. 6D). As shown in the figure, blue represents the timeline, red represents the time period highlighted by the keyword. The time period shows the year from the outbreak to the end of the keyword and its duration, and strength represents the intensity of the outbreak.^[4]^ Hla-b27 is the strongest, followed by heavy chain, class i molecule, spondyloarthritide, systemic lupus erythematosus. In addition, a review of the articles reveals the historical evolution of research hotspots from 2004 to 2023, and it can be seen that the main lines of research in the field of B cells intervention in inflammatory mechanism of AS have changed over time.

## 4. Discussion

### 4.1. General information

We searched the WOS database from 2004 to 2023 for published journals articles and dissertations on B cells intervention in inflammatory mechanism of AS. CiteSpace 6.1.R6 software was used to conduct the visualization analysis of countries, authors, institutions, references and keywords, and totally collected 359 related articles.

Since 2004, the number of published papers in the field of B cells intervention in inflammatory mechanism of AS has fluctuated and increased, indicating that the field has made considerable development and progress in the past 20 years. During the rapid increase in publications from 2007 to 2009, Baeten, D et al raised the question of whether B cells could be future therapeutic targets for AS.^[7]^ It can be seen that in the early stage of the development of this field, literature studies were still mainly focused on raising clinical questions, and researchers gradually realized that B cells may play an important role in the process of AS inflammation. The number of published papers gradually decreased from 2017 to 2020. During this period, the number of experimental and clinical studies was small, and theoretical studies accounted for the majority, which may be related to the lack of research funding caused by the novel coronavirus epidemic, proving that appropriate support is needed in the critical period of the development of emerging research fields. In 2021, when the number of papers reached its peak, a large number of research results emerged, proving that the research value of B cells intervention in inflammatory mechanism of AS has been fully valued by researchers and put into practice.

China, the United States and Germany are the countries with the largest number of published literatures and the highest level of international cooperation, indicating that scientists from these 3 countries play a leading role in the study of B cells intervention in inflammatory mechanism of AS. Except for China, the top 10 countries in the number of published papers are all developed countries, which indicates that developed countries have made greater contributions to this research field while China is at the forefront of developing countries. Therefore, it is very necessary to strengthen international cooperation and exchanges. Masters, SL et al defined 6 types of autoinflammatory diseases, including complement regulation diseases and cytokine signaling disorders,^[8]^ which was the most cited (766 citations) in the US study on the mechanism of B cells intervention in AS inflammation. It shows that American scholars focus on exploring how B cells intervene in autoinflammatory diseases such as AS by secreting complement and cytokines. Zhang, L et al found that Th22 cells and Th17 cells may be related to the pathogenesis of AS,^[9]^ which is the most cited article in China (196 citations), indicating that the main research direction of Chinese scholars is to prove that the pathogenesis of AS is not independently dominated by B cells.

The top 10 most productive authors have a total of more than 60 articles, and the top 3 are Bowness, Paul, Breban, Maxime, and Cauli, Alberto, and they form a close-knit community around these 3 authors, most of whom are affiliated with the same research institution as their coauthors. One of the most widely published papers is Bowness Paul, whose research group works closely with other groups and is very influential in this field. His research focused on how B cells collaborate with other immune cells to intervene in AS inflammation, demonstrating that endoplasmic reticulum aminopeptidase 1 plays a key role in determining HLA-B27 FHC expression and Th17 response levels in AS, which in turn enables B cells to fully activate. The 2 can influence each other to jointly mediate the inflammatory immune response of AS.^[10]^

Anhui Med Univ, Charite and Free Univ Berlin are leading institutions in the study of B cells intervention in inflammatory mechanism of AS, and have carried out influential regional cooperation in China, Germany and Spain respectively. An article by Renfang Han et al in Anhui Medical University revealed that CD8 + T cells may be involved in regulating the inflammation level of AS patients through its cytotoxic effect combined with the antigen presentation of B cells, thus inducing the autoimmune response of AS, so as to interfere with the pathogenesis and progression of AS.^[11]^

The higher the citation frequency, the higher the influence or importance of a particular article, and these documents have been co-cited many times, which can be regarded as a “knowledge base” by other authors in the field.^[5]^ According to the results of co-cited literature analysis, the current research on the mechanism of B cells intervention in AS inflammation mainly focuses on “intergenic region,” “bone mineral density” and “potential place.” Ansalone, C and their coauthors used the HLA-B27 transgenic rat model to prove that intestinal inflammation in the pathogenesis of AS has the potential to directly drive systemic inflammation and bone erosion in which immune cells such as B cells participate, and that peripheral environmental stimulation is also an important influencing factor for B cells to intervene in the inflammatory response of AS.^[12]^

According to the results of keyword cluster analysis, the different categories can be divided into 4 categories: endoplasmic reticulum secreted proteins participate in the construction of AS inflammatory signal pathway, other cells cooperate with B cells to participate in the inflammatory mechanism of AS, B cells participate in the inflammatory response mechanism of other diseases, and the recognition mechanism of AS immune response, which proves that the research content in this field is not limited to the intervention of B cells in the inflammatory mechanism of AS. Other cells involved in the inflammatory response of AS and the intervention of B cells in the inflammatory response mechanism of other diseases have also been extensively studied, and the research results show that many immune cells such as B cells participate in the inflammatory response of AS and other autoimmune diseases and influence each other.^[13]^

### 4.2. Research hotspots/frontiers

From the results of keyword co-occurrence, emergence and time graph, the current research in this field mainly focuses on the cellular immune mechanism of B cells intervention in inflammatory mechanism of AS, the humoral immune mechanism of B cells intervention in inflammatory mechanism of AS, and the clinical application value of B cells intervention in inflammatory mechanism of AS.

### 4.3. The cellular immune mechanism of B cells intervention in inflammatory mechanism of AS

B cells play an important role in the inflammatory mechanism of AS and other autoimmune diseases, and can promote the autoimmune response of AS by acting as specialized antigen-presenting cells and other mechanisms.^[14]^ CD8 + CD122 + T cells are a newly discovered natural immune regulatory T cells with negative immune regulatory function, and their importance in maintaining immune homeostasis has been fully demonstrated by some animal disease models.^[15]^ CD8 + CD122 + T cells may also secrete excessive amounts of cytokines such as interleukin 10(IL-10) and IL-15. In conclusion, the imbalance of regulatory T cells may lead to immune system dysfunction in chronic autoimmune diseases such as AS. In addition, research results show that CD8 + T cells may participate in the pathogenesis and progression of AS and are expected to become a new target to regulate the inflammation level of AS patients, while activated B cells can present intracellular and extracellular antigens to CD8 + T cells, prompting them to differentiate into cytotoxic T lymphocyte cells and activate cytotoxic effects, leading to chronic inflammation or directly acting on cell killing. The corresponding pathological damage to the own tissues is caused.^[11]^

In addition, B cells can promote disease inflammation through presentation of autoantigens and subsequent activation of T lymphocytes. Studies have shown that the role of B cells as antigen-presenting cells is enhanced when the antigen peptides presented by B cells are identical to those that bind to the antibodies they produce. The homologous recognition of the same antigen by B cells and T cells enables mutual activation between them. T cells activate B cells to express costimulatory molecules CD 80 and CD 86. B cells assist in activating T cells to differentiate into follicular helper T cells that produce IL-4, IL-5, IL-10 and IL-13. IL-4 can support CD40-induced amplification of memory B cells and class switch recombination to produce immunoglobulin G4 or IgE, while IL-21 can differentiate antigen-activated B cells into high-affinity plasma cells to produce specific antibodies to mediate AS inflammatory immune response.^[10]^

### 4.4. The humoral immune mechanism of B cells intervention in inflammatory mechanism of AS

B cells can promote the inflammatory response of AS and other autoimmune diseases through various humoral immune mechanisms, including the production of autoantibodies and the release of cytokines.^[7]^ B cells develop from common lymphoprogenitor cells in a gradual manner, and this process transitions through B cell development checkpoints. If the checkpoints do not eliminate the autoreactive B cells, defects in their development and maturation will result in the production of autoantibodies.^[16]^Autoantibodies are secreted immunoglobulins of the same type as IgM, IgG, IgA, or IgE.^[17]^ Although specific autoantibodies have not been identified in human AS, B cells and plasma cells that produce autoantibodies after terminal differentiation of B cells are abundant in inflammatory peripheral joints and axial skeleton.^[7]^ Studies have shown that autoantibodies can directly promote the progression of AS through innate and adaptive immune mechanisms, ultimately leading to systemic inflammation and local tissue damage. In addition, genetic variation, environmental triggers and antigen modification involved in the regulation of B cell tolerance are also key mechanisms for the development of autoantibodies of AS.^[18]^ The genetic variation includes the molecule of antigen presentation mechanism, which is associated with specific human leukocyte antigen(HLA) class II haplotype and several non-HLA genes.^[19]^ Environmental triggers include exposure to different toxic substances and infection by pathogens associated with autoantibody responses such as bacteria or viruses.^[20]^ Antigenic modification can also alter the immunogenicity of self-protein molecules, thus leading to the recognition of self-reactive B cells.^[21]^ All of the above factors may lead to impaired B cell tolerance and systemic inflammation and local tissue damage by promoting AS to produce autoantibodies.

In addition, B cells can also promote the inflammatory response of autoimmune diseases such as AS by releasing different cytokines. The experiment shows that IL-5 produced by CD10 + B cells at mRNA and protein levels is one of the main effecting functions of Breg cells in different experimental models. In addition, CD5 + B cells can also produce higher levels of IL-6, indicating the plasticity and complexity of B cell function.^[22]^ Studies have shown that IL-5 can promote the differentiation of antigen-stimulated B cells into antibody synthesizing cells, mainly act on B cells entering the late stage of cell proliferation, and increase the expression of IL-2R in activated B cells. The stimulating effect of IL-5 is similar to the function of IL-6, and only acts in a very narrow phase after B cell stimulation. In addition, IL-5 may promote IgA synthesis by acting as an IgA specific promoter to differentiate mIgM positive B cells into mIgA positive B cells and by acting on B cells of IgA type to promote their proliferation and differentiation into plasma cells that secrete IgA. IL-5 also promotes the secretion of IgM.^[23]^ By producing the above cytokines, B cells promote the proliferation and differentiation of own cells and the secretion of antibodies, mediating the inflammatory response of AS and other autoimmune diseases.

### 4.5. The clinical application value of B cells intervention in inflammatory mechanism of AS

Research finds that the percentage of CD19 + B cells in AS patients is positively correlated with the scores of bath AS disease activity index, patient global assessment, visual analogue scale and nocturnal visual analogue scale, indicating that B cells and peripheral blood subgroups in active AS patients and peripheral joint affected patients are out of balance. Therefore, it is believed that B cells may play an important role in the inflammatory mechanism of AS.^[24]^ In addition, the presence of B cells in peripheral and axial lesions in patients with AS has been identified, raising the question of whether B lymphocytes can also be appropriate therapeutic targets for AS, and providing evidence through clinical observation that active AS can occur in the absence of functionally mature B cells. Therefore, the need to systematically study the exact role and function of B lymphocytes in this disease is emphasized.^[7]^ Membrane glucocorticoid receptor(mGCR) is a nuclear transcription factor on cell membrane, widely existing in various tissues and cells of the body. After binding with glucocorticoid, mGCR regulates gene transcription by binding to a specific DNA sequence on the target gene in the nucleus, and exerts various biological effects. The expression of mGCR on peripheral blood mononuclear cells of patients with AS is experimentally studied, which shows that mGCR is upregulated in B cells from AS patients. This upregulation does not correlate with the immoral or overall disease activity, which may be related to the ability of low-dose glucocorticoid to delay the inflammatory process to some extent, and the ability of high-dose (intravenous or intra-articular) glucocorticoid to rapidly and efficiently inhibit the inflammatory process in AS. Therefore, selective use of drugs that can bind to mGCR may be a new option for the treatment of AS.^[25]^

## 5. Limitation

There are some limitations to the study. Firstly, all the data in this study are obtained from the WOS database, and articles published in other databases may be ignored. Secondly, this study only includes English articles, excluding the articles of other languages, which may lead to some limitations in the scope of the study. Thirdly, the literature in the WOS database is constantly being updated, and the number of literature and the information included in the literature may change with time, which may lead to bias in the final results. Finally, some recently published high-quality articles may not have received our attention due to insufficient time to be cited.^[4]^

## 6. Conclusion

Over the past 20 years, research on the mechanism by which B cells intervene in AS inflammation has fluctuated globally. China, the United States and Germany play a leading role in this field, and it is necessary to strengthen international exchanges and cooperation. Current studies focus on B cells’ ability to intervene in AS inflammation through the humoral immune mechanism of producing autoantibodies and secreting cytokines, and the cellular immune mechanism jointly mediated by Th cells and CD8 + T cells. Future research hotspots may tend to use B cells as a new therapeutic target for AS, and the selective use of drugs that can bind mGCR may become a new choice for AS treatment. Given the relatively new field of research, the lack of high-quality prospective clinical randomized controlled trial, and the small sample size of experimental studies, there is a need to scale up clinical research in the future and to strengthen multi-center collaboration and communication, in order to promote the transformation and application of research results.^[5]^

## Acknowledgments

We thank all authors who participated in the study of B cells intervention in inflammatory mechanism of AS.

## Author contributions

**Conceptualization:** Qing Yu.

**Data curation:** Qing Yu, Zhaoyi Liu.

**Formal analysis:** Zhaoyi Liu.

**Funding acquisition:** Hongxiao Liu.

**Investigation:** Qing Yu.

**Methodology:** Qing Yu, Xiaohan Xu.

**Project administration:** Xiaohan Xu, Hongxiao Liu.

**Software:** Qing Yu.

**Supervision:** Hongxiao Liu.

**Validation:** Xiaohan Xu.

**Writing – original draft:** Qing Yu, Zhaoyi Liu.

**Writing – review & editing:** Hongxiao Liu.
